# Supplementary material for: Novel Insights to Assess Climate Resilience in Goats Using a Holistic Approach of Skin-Based Advanced NGS Technologies
Source: Int J Mol Sci. 2023 Jun 19;24(12):10319. doi: 10.3390/ijms241210319 (PMC10298993; doi:10.3390/ijms241210319)
Supplement: Supplementary file 1 [file ijms-24-10319-s001.zip › ijms-2451291-supplementary.pdf]

Supplementary figures:

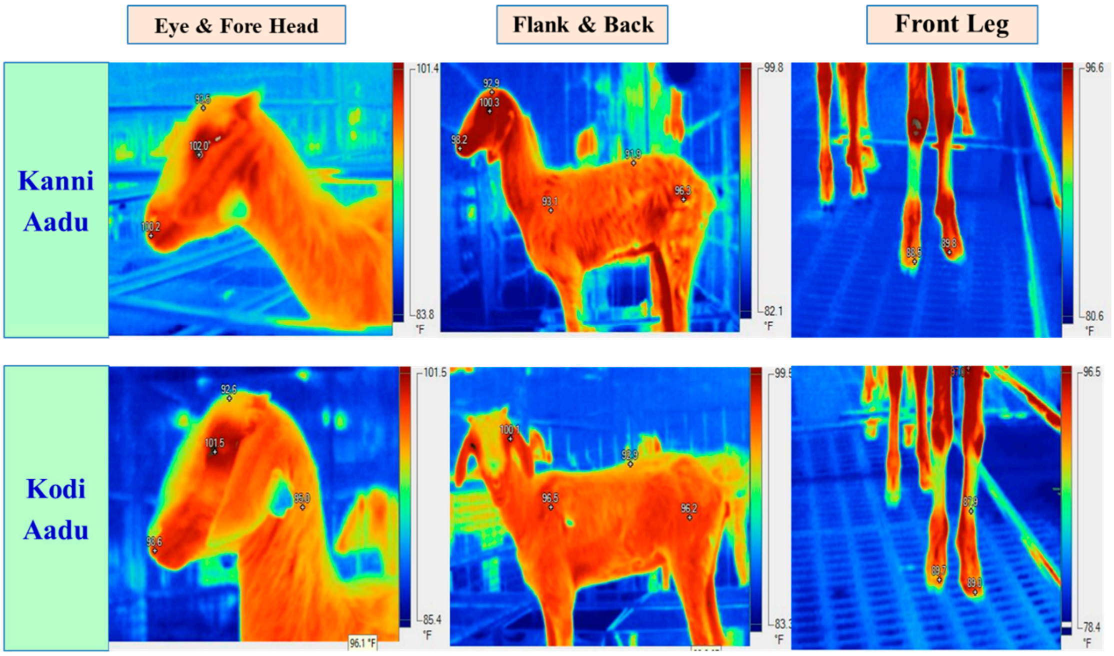

Figure S1. Infrared thermal images of Kanni Aadu and Kodi Aadu goats during heat stress

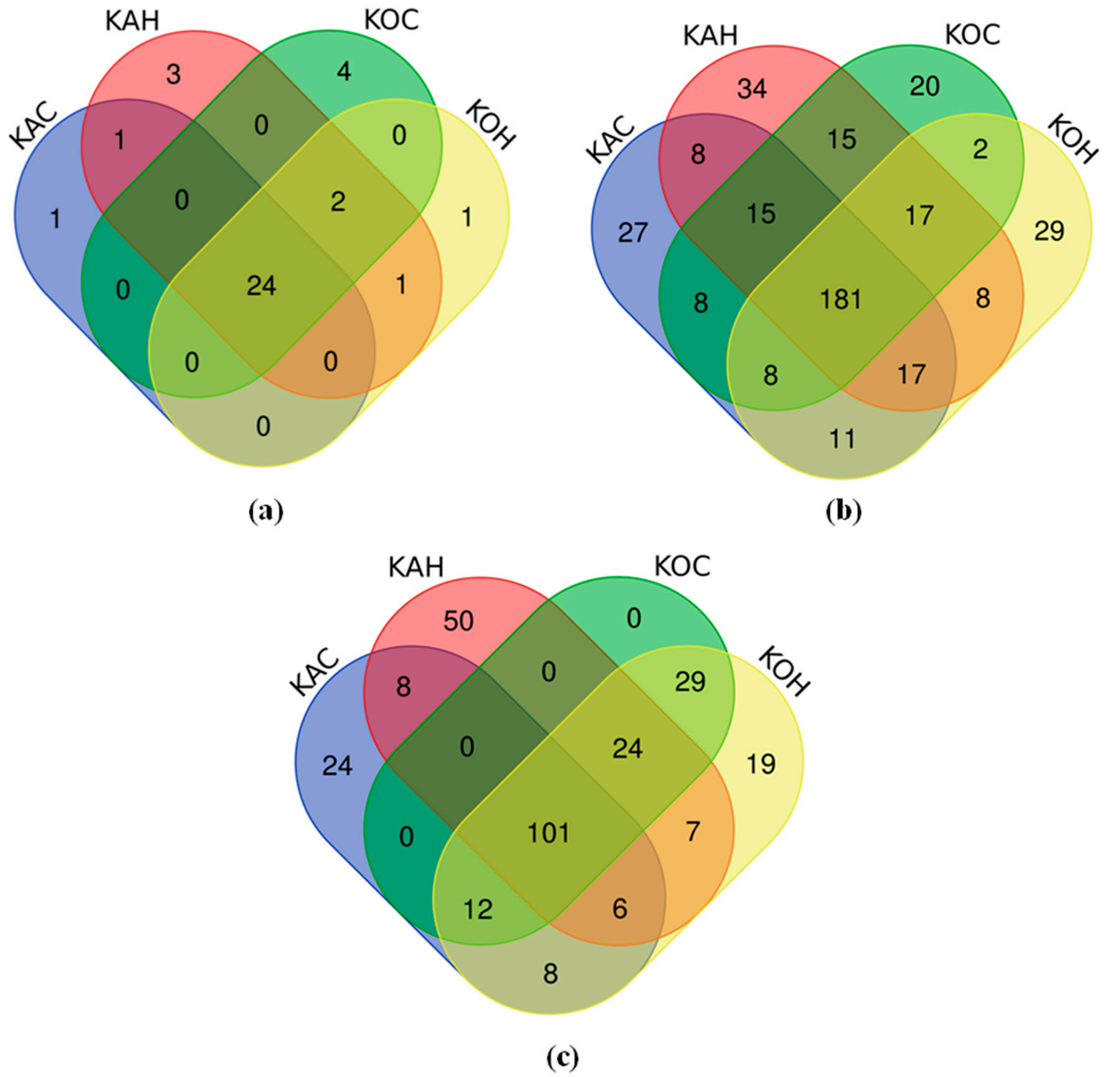

Figure S2. Venn diagram depicting the phylum (a), genus (b) and species (c) level skin microbiome unique and common between the 4 experimental groups (KAC- Kanni Aadu control; KAH- Kanni Aadu heat stress; KOC- Kodi Aadu control; KOH- Kodi Aadu heat stress). The number of unique species in each group has been depicted while those in the overlapping areas are the number of common taxa between the groups.

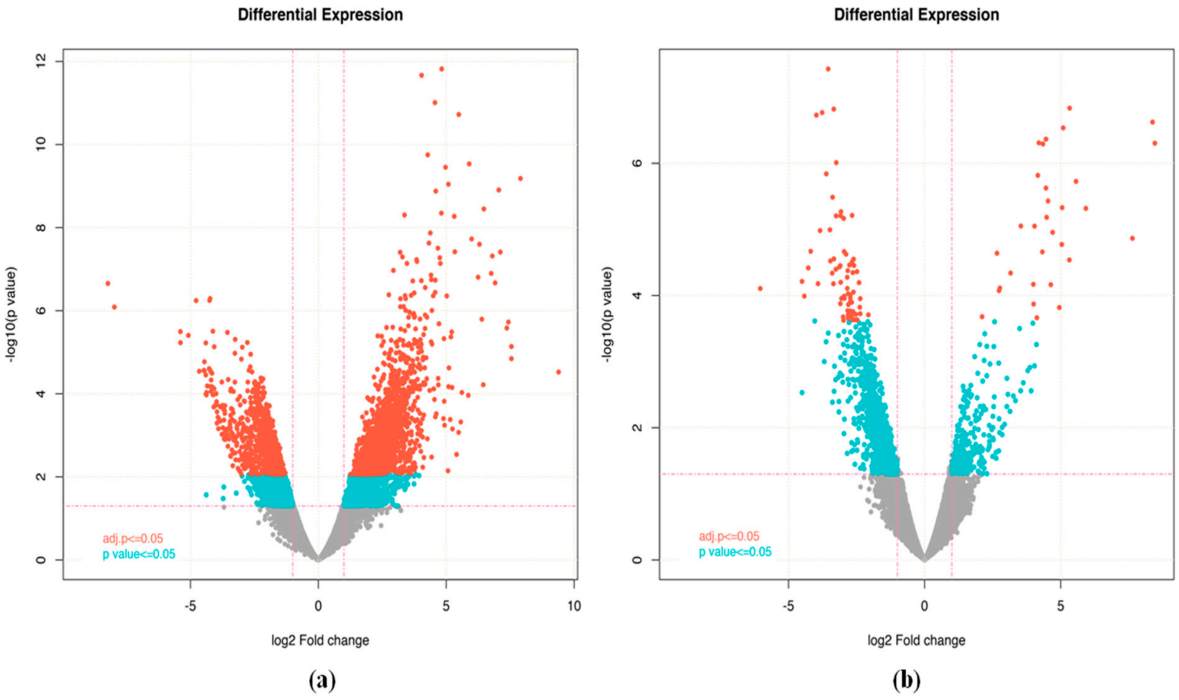

Figure S3. Volcano plots depicting the overall DEGs expressed in KAC_vs_KAH (a) and KOC_vs_KOH (b). The significantly expressed DEGs are depicted in blue ($p<=0.05$) and orange (adj.$p<=0.05$) dots.

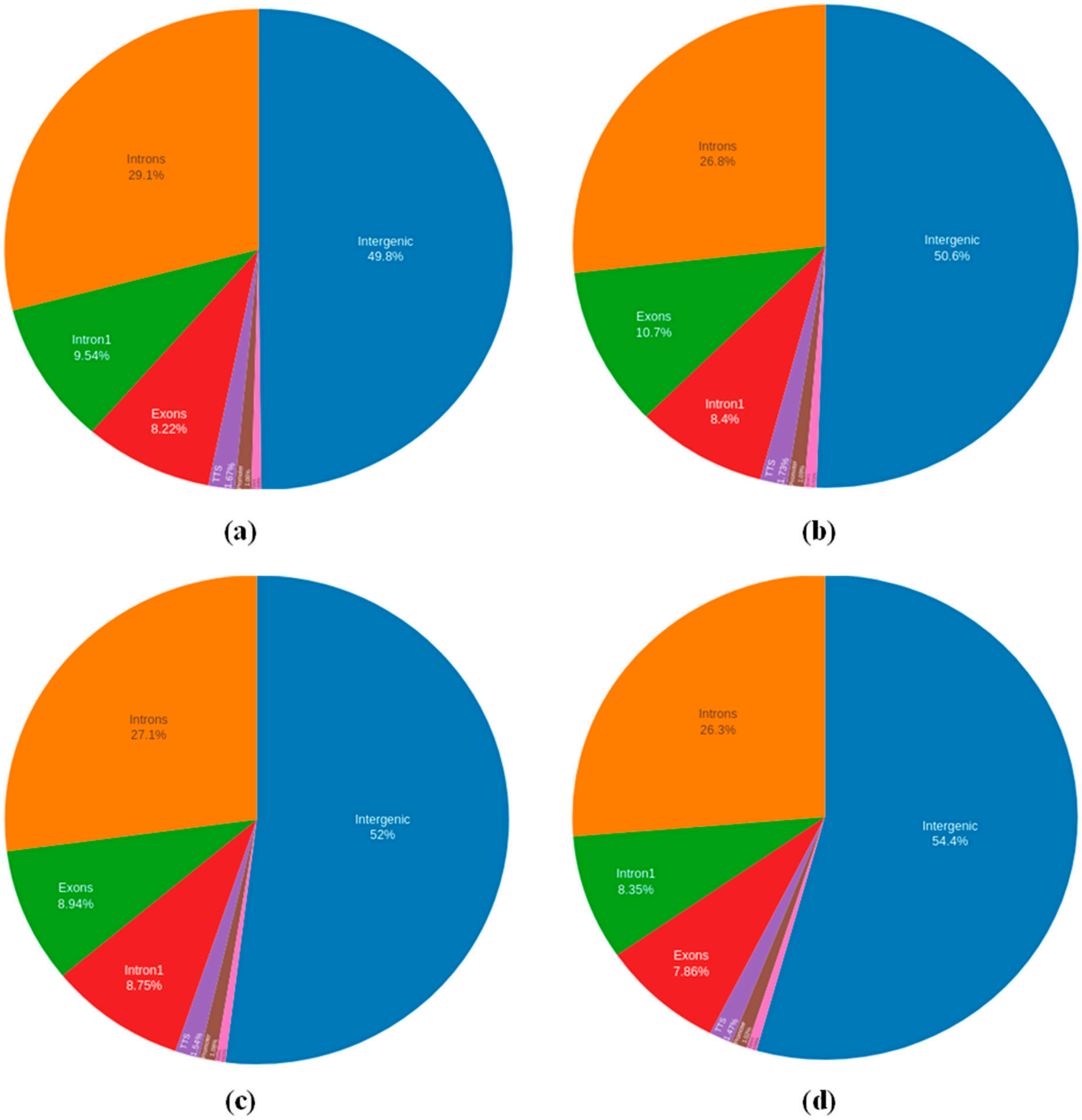

Figure S4. Overview of hypermethylation at the CGI (CG islands) regions and its distribution across the genomic functional regions in KAC (a), KAH (b), KOC (c) and KOH (d).

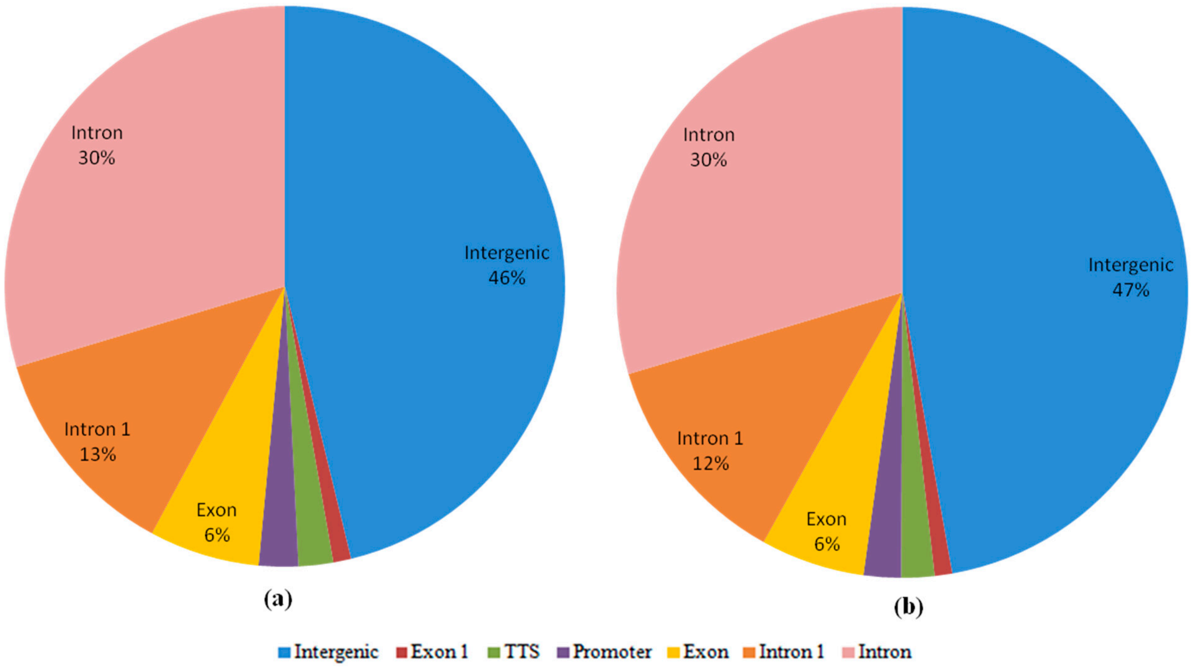

Figure S5. Overview of distribution of differentially methylated regions across the genomic functional regions in KAC_vs_KAH (a) and KOC_vs_KOH (b)

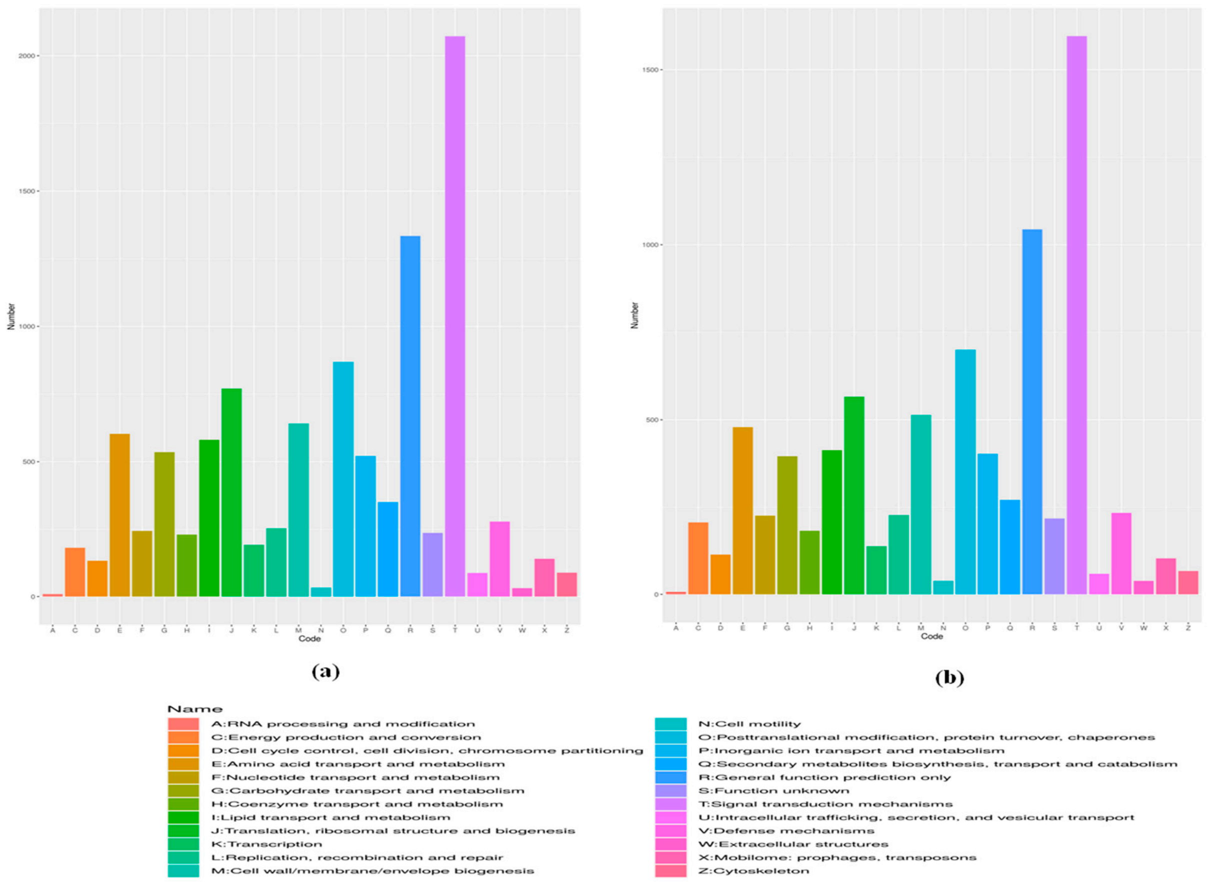

Figure S6. Functional analysis predicting the clusters of orthologous groups of proteins (COGs) of the CpG DMRs as a consequence of heat stress in KAC_vs_KAH (a) and KOC_vs_KAH (b)

Supplementary tables:

Table S1. Alpha diversity indices of skin microbiome in Kanni Aadu and Kodi Aadu goats

	**Chao1**	**ACE**	**Shannon**	**Simpson**	**InvSimpson**	**Fisher**
KAC	804.53±56.35	754.45±14.42	3.74	0.95	20.19	82.01
KAH	818.32±43.85	786.90±14.31	4.23	0.97	32.71	96.84
KOC	666.17±27.27	669.43±13.02	4.06	0.96	26.98	83.20
KOH	711.11±44.36	681.39±13.31	4.22	0.97	31.85	78.86
p-value	0.76	0.76	0.60	0.17	0.17	0.84

Table S2. Read alignment statistics of Kanni Aadu and Kodi Aadu goat skin tissue subjected to transcriptomics

Group	Number of reads (in million)	Reads after QC (in million)	Mapped reads (in million)	Mapped reads %	Uniquely mapped reads %	Unmapped Reads %
KAC	45.01	39.57	32.90	83.23	60.82	16.77
KAH	37.74	33.95	31.82	93.78	74.95	6.22
KOC	40.50	35.48	30.24	86.54	67.17	13.46
KOH	39.28	35.53	29.31	82.55	61.12	17.45

Table S3. Top 20 significantly expressed DEGs in Kanni Aadu and Kodi Aadu goat skin due to heat stress

KAC_vs_KAH		KOC_vs_KOH	
**Prdicted gene names (gene symbol)**	**Log2FC**	**Prdicted gene names (gene symbol)**	**Log2FC**
Hemoglobin fetal subunit beta (LOC102176710)	9.39	Ubiquitin carboxyl-terminal hydrolase 17-like protein 6 (LOC108634750)	8.38
Secretoglobin family 2B member 20 (LOC102168380)	7.90	Major allergen I polypeptide chain 1-like (LOC102169411)	5.56
Major allergen I polypeptide chain 2 (LOC102169125)	7.55	Major allergen I polypeptide chain 2 (LOC102169695)	5.33
Major allergen I polypeptide chain 1 (LOC106503120)	7.55	Major allergen I polypeptide chain 2 (LOC102169125)	5.32
Major allergen I polypeptide chain 1-like (LOC102169411)	7.43	Ubiquitin carboxyl-terminal hydrolase 17-like protein 6 (LOC108635524)	5.09
Major allergen I polypeptide chain 1-like (LOC108637981)	7.37	Major allergen I polypeptide chain 1-like (LOC108637981)	5.05
Odorant-binding protein-like (LOC108634312)	7.12	Major allergen I polypeptide chain 1 (LOC106503120)	5.04
Protein S100-A7A (S100A7A)	7.06	Ubiquitin carboxyl-terminal hydrolase 17-like protein 6 (LOC108635523)	4.95
Protein S100-A7-like (LOC108633164)	6.91	Major allergen I polypeptide chain 1-like (LOC102191166)	4.70
Major allergen I polypeptide chain 2 (LOC102169695)	6.80	E3 ubiquitin-protein ligase SIAH1-like (LOC108634445)	4.64
Olfactory receptor 1L8-like (LOC102168271)	-8.23	Hemoglobin subunit beta-like (LOC102182615)	-6.04
Ubiquilin-3 (UBQLN3)	-7.97	Carbonic anhydrase 1-like (LOC108635261)	-4.51
Olfactory receptor 1K1 (LOC102184531)	-5.39	Olfactory receptor 51I1-like (LOC102173673)	-4.50
Olfactory receptor 1J4-like (LOC108637117)	-5.08	39S ribosomal protein L51, mitochondrial (MRPL51)	-4.42
Potassium voltage-gated channel subfamily E member 2 (KCNE2)	-4.78	Hemoglobin subunit zeta-like (LOC108633873)	-4.28
Olfactory receptor 2L2-like (LOC102191036)	-4.48	Filaggrin-2-like isoform X1; filaggrin-2-like isoform X2 (LOC108634870)	-4.19
Apolipoprotein L2-like (LOC108636108)	-4.45	Keratin-associated protein 12-2 (LOC102189437)	-3.98

Putative ATP-dependent RNA helicase TDRD12, partial (LOC108634865)	-4.45	Loricrin (LOR)	-3.92
Pregnancy-associated glycoprotein 1-like (LOC102169373)	-4.40	Retroviral-like aspartic protease 1 (ASPRV1)	-3.84
Hair acidic keratin 1 (LOC100861381)	-4.38	Keratin-associated protein 9-2-like (LOC108638292)	-3.77

Table S4. Overview of DEGs associated with some of the heat stress associated GO pathways in Kanni Aadu and Kodi Aadu goat skin on heat stress exposure

Kanni Aadu		Kodi Aadu	
**Gene Name (gene ID)**	**Log2FC**	**Gene Name (gene ID)**	**Log2FC**
Oxygen transporter activity [MF: GO:0005344]		Structural molecule activity [MF: GO:0005198]	
HBBC (102175045)	5.57	LOC100861381	-3.15
LOC102174495	5.49	KRT25 (100861172)	-2.99
LOC108633874	4.92	KRT27 (100861382)	-2.94
LOC102168959	5.86	KRTAP3-1 (100861180)	-2.93
MB (100860833)	-1.95	LOC100861179	-2.43
LOC102175876	3.37		
LOC102176710	9.39		
NADH dehydrogenase (ubiquinone) activity [MF: GO:0008137]			
ND2 (1485857)	3.76	ND2 (1485857)	-2.26
ND1 (1485856)	3.17	ND1 (1485856)	-1.87
ND6 (1485867)	2.64	ND6 (1485867)	-2.02
ND5 (1485866)	3.17	ND5 (1485866)	-2.54
NDUFB8 (100861349)	1.96	NDUFB8 (100861349)	-1.89
NDUFS7 (100860782)	2.70	ND4L (1485864)	-2.03
ND4 (1485865)	3.41		
ND4L (1485864)	3.86		
ND3 (1485863)	3.02		
		Intermediate filament [CC: GO:0005882]	
		LOC100861175	-2.53
		KRTAP15-1 (100861176)	-3.01
		LOC100861181	-3.08
		KAP8 (100861182)	-2.23
		LOC100861381	-3.15
		KRT25 (100861172)	-2.99
		KRT27 (100861382)	-2.94
		KRTAP11-1 (100861173)	-3.13
		LOC100860930	-3.04
		Keratin filament [CC: GO:0045095]	
		LOC100861174	-2.62
		LOC100861184	-2.77
		KRTAP3-1 (100861180)	-2.93
		LOC100861179	-2.43
		Mitochondrion [CC: GO:0005739]	
		ND2 (1485857)	-2.26
		CYTB (1485868)	-2.14
		LOC100861197	-1.40
		APEX1 (100860967)	-1.71
		ND6 (1485867)	-2.02
		ND5 (1485866)	-2.54
		ND4L (1485864)	-2.03

Table S5. Simulated heat stress model used to induce heat stress in KAH and KOH group goats housed in the heating chamber of the climate chamber

Time	10:00 am	11:00 am	12:00 pm	01:00 pm	02:00 pm	03:00 pm	04:00 pm
Temperature	36 ºC	37.5 ºC	38.5 ºC	38.8 ºC	40 ºC	38.3 ºC	37 ºC
Relative Humidity	49%	44%	41%	40%	39%	42%	45%
